# Multi-country collaborative citizen science projects to co-design cardiovascular disease prevention strategies and advocacy: findings from Ethiopia, Malawi, Rwanda, and South Africa

**DOI:** 10.1186/s12889-023-17393-x

**Published:** 2023-12-12

**Authors:** Kufre J. Okop, Kiya Kedir, Stephen Kasenda, Jean Berchmans Niyibizi, Effie Chipeta, Hailemichael Getachew, Kerstin Sell, Estelle Victoria Lambert, Thandi Puoane, Stephen Rulisa, Christopher Bunn, Abby C. King, Charlotte Bavuma, Rawleigh Howe, Amelia C. Crampin, Naomi S. Levitt

**Affiliations:** 1 Chronic Disease Initiative for Africa, Department of Medicine, University of Cape Town, South Africa Cape Town,; 2 Armauer Hansen Research Institute (AHRI), Addis Ababa, CA Ethiopia; 3 Malawi Epidemiology and Intervention Research Unit, Lilongwe, Malawi, Lilongwe, Malawi; 4 Directorate of Research and Innovation, College of Medicine and Health Sciences, University of Rwanda, Kigali, Rwanda; 5 Centre for Reproductive Health, College of Medicine, University of Malawi, Blantyre, Malawi; 6 Chair of Public Health and Health Services Research, IBE, Faculty of Medicine, LMU Munich, Germany; 7 UCT Research Centre for Health Through Physical Activity, Lifestyle and Sport, Division of Exercise Science and Sports Medicine, Faculty of Health Sciences, University of Cape Town, Cape Town, South Africa; 8 School of Public Health, University of the Western Cape, Cape Town, South Africa; 9 School of Medicine and Pharmacy, College of Medicine and Health Sciences, College of Medicine and Health Sciences, University of Rwanda, Kigali, Rwanda; 10 College of Social Sciences, University of Glasgow, Glasgow, UK; 11 Department of Epidemiology and Population Health, Stanford University School of Medicine, Stanford, USA CA; 12 Department of Medicine (Stanford Prevention Research Center), Stanford University School of Medicine, Stanford, USA CA; 13 Department of Population Health, London School of Hygiene & Tropical Medicine, London, UK; 14 Pettenkofer School of Public Health, Munich, Germany; 15 Citizen Science Research Foundation (CSRF), Cape Town, South Africa

**Keywords:** Citizen Science, Community-based, Co-design, Cardiovascular disease, Prevention strategies, Advocacy, Participatory, Sub-Saharan Africa

## Abstract

**Background:**

Cardiovascular diseases (CVD) were responsible for 20.5 million annual deaths globally in 2021, with a disproportionally high burden in sub-Saharan Africa (SSA). There is growing evidence of the use of citizen science and co-design approaches in developing interventions in different fields, but less so in the context of CVD prevention interventions in SSA. This paper reports on the collaborative multi-country project that employed citizen science and a co-design approach to (i) explore CVD risk perceptions, (ii) develop tailored prevention strategies, and (iii) support advocacy in different low-income settings in SSA.

**Methods:**

This is a participatory citizen science study with a co-design component. Data was collected from 205 participants aged 18 to 75 years in rural and urban communities in Malawi, Ethiopia and Rwanda, and urban South Africa. Fifty-one trained citizen scientists used a mobile app-based (EpiCollect) semi-structured survey questionnaire to collect data on CVD risk perceptions from participants purposively selected from two communities per country. Data collected per community included 100–150 photographs and 150–240 voice recordings on CVD risk perceptions, communication and health-seeking intentions. Thematic and comparative analysis were undertaken with the citizen scientists and the results were used to support citizen scientists-led stakeholder advocacy workshops. Findings are presented using bubble graphs based on weighted proportions of key risk factors indicated.

**Results:**

Nearly three in every five of the participants interviewed reported having a relative with CVD. The main perceived causes of CVD in all communities were *substance use*, *food-related factors*, and *litter*, followed by *physical inactivity*, *emotional factors, poverty, crime*, and *violence*. The perceived positive factors for cardiovascular health were *nutrition, physical activity*, *green space*, and *clean/peaceful communities*. Multi-level stakeholders (45–84 persons/country) including key decision makers participated in advocacy workshops and supported the identification and prioritization of community-specific CVD prevention strategies and implementation actions. Citizen science-informed CVD risk screening and referral to care interventions were piloted in six communities in three countries with about 4795 adults screened and those at risk referred for care. Health sector stakeholders indicated their support for utilising a citizen-engaged approach in national NCDs prevention programmes. The citizen scientists were excited by the opportunity to lead research and advocacy.

**Conclusion:**

The collaborative engagement, participatory learning, and co-designing activities enhanced active engagement between citizen scientists, researchers, and stakeholders. This, in turn, provided context-specific insights on CVD prevention in the different SSA settings.

## Background

Death due to cardiovascular disease (CVDs) rose globally by 60% from 12.1 million in 1990 to 20.5 million in 2021, with nearly four out of five of these occurring in low-income countries, including sub-Saharan Africa (SSA) [1, 2]. There are considerable knowledge and capacity gaps in CVD services delivery in many African settings [3–5]. In the SSA region, uptake of population-based CVD risk screening and prevention interventions by socio-economically poor populations are hampered by the generally low levels of knowledge and awareness of CVD and associated risk factors, and the often inaccurate perceptions of severity of risk [5]. Qualitative studies in SSA settings have shown that community perception and understanding of the concept of CVD risk could be a barrier to the uptake of population-based CVD risk prevention interventions including screening and care [6–8]. A recent qualitative meta-synthesis reported a disconnect between daily lived experiences of African people and perception of CVD, its risk factors, and indicated that treatment options were influenced by religious and cultural factors [9].

Innovative interventions that take varying local contexts into consideration and embrace the participation of communities and collaboration with relevant stakeholders are needed to support CVD prevention in low- and middle-income countries (LMICs) [4, 10]. Indeed, community-based intervention projects that take a grounded, co-design approach where local community members, stakeholders, and scientists participate in research are known to increase results-oriented participation in science and enable co-production of sustainable solutions [11–13]. Furthermore, participatory population-based intervention programmes that effectively engage and train local citizens as scientists and foster collaboration and solution-building across social and environmental structures have had substantial impact on community health [14]. While there is a growing evidence of the use of community-based participatory and co-design approaches in developing and implementing interventions in different populations, there is limited evidence of the use of this approach in the prevention of CVD in multiple settings in LMICs, and globally [15–17]. Co-design is a participatory approach that brings together implementers and beneficiaries to design local solutions to address local problems. Often the terms ‘co-design’, ‘co-creation’, and ‘co-production’ are used interchangeably to describe the development of interventions that involves multiple stakeholders [18].

Recent systematic and scoping reviews have indicated the appropriateness of using a codesign or co-production approach to designing health-related programmes [17, 19], and to promote health policies focusing on CVD prevention [20]. Citizen science and co-design approaches have been used to facilitate stakeholders’ engagement, participatory learning, adaptation of tools/processes, co-creation of knowledge, and advocacy for social action [21, 22]. As part of a research task under a larger CEBHA + (Collaboration for Evidence-based Health Care and Public) consortium [23], we set out to implement and evaluate a multi-country community-engaged citizen science study to which aimed to explore CVD risk perception, and develop tailored prevention strategies and support advocacy in low-income settings in SSA [24].

**Fig. 1:**
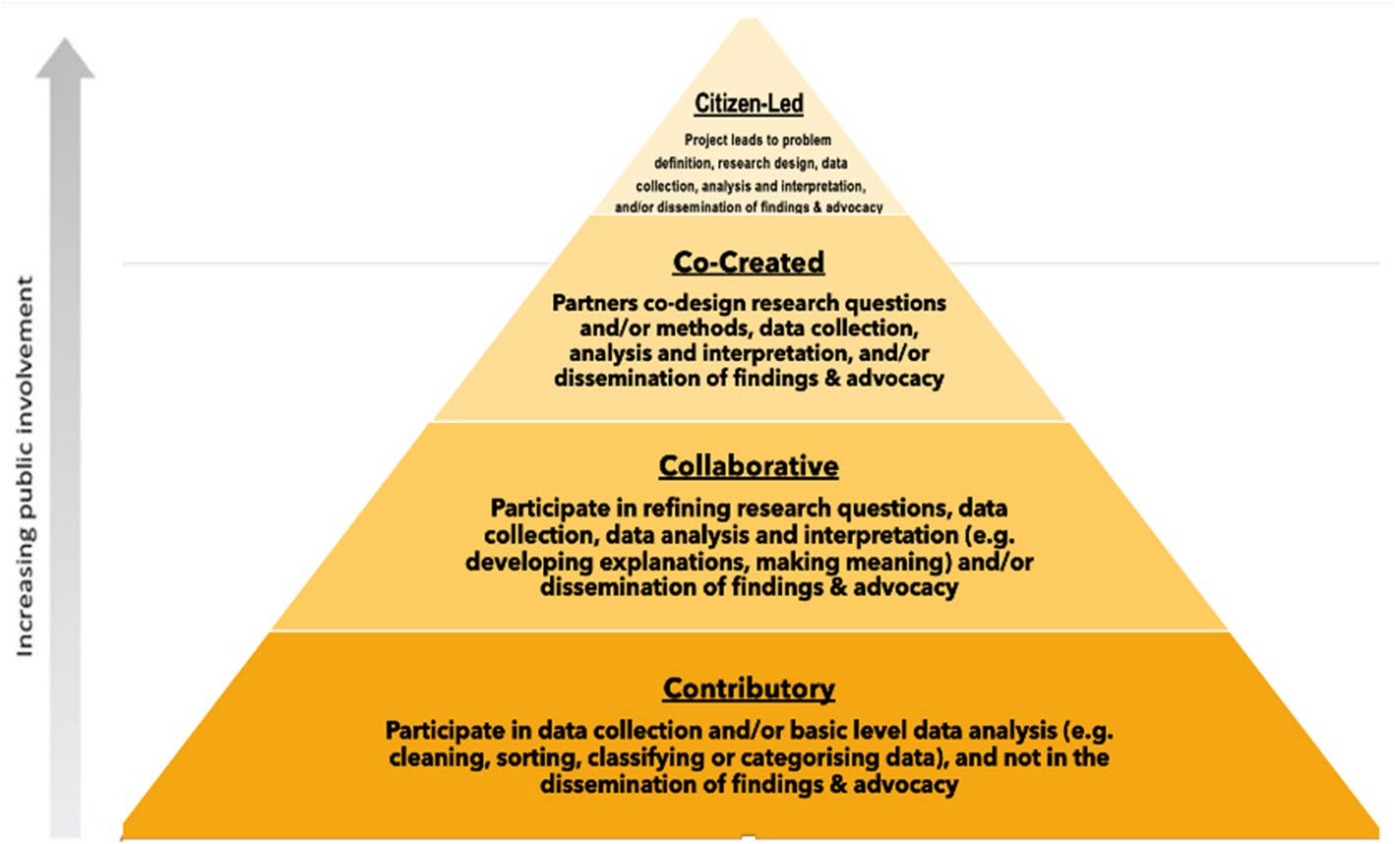
Four models (framework) of citizen science characterized by increasing levels of public involvement in the research process. Adapted from Den Broeder et al. 2018, [22], and Marks et al., 2022 [15]

A citizen science framework has been described by Den Broeder et. al. (2018), and further adapted by Marks and colleagues [15] (See Fig. 1) to include four main participatory approaches, viz. *contributory, collaborative*, *co-created,* and *citizen-led* approaches. A recent systematic review has reported the use of this framework in supporting predominantly small-scale co-produced physical activity and nutrition related projects directed at the prevention of chronic diseases mainly in countries outside of Africa [15]. Our research falls generally into the “collaborative” category of the citizen science framework as seen in Fig. 1. This paper reports on the adaptation, outcomes, challenges, and lessons learnt in implementing collaborative CVD risk prevention research project, using citizen science and co-design approach in the urban and rural settings in four SSA countries.

### Study goal and objectives

This study documents the implementation and outcomes of a multi-country collaborative project that employed citizen science and co-design approach to explore CVD risk perceptions, and develop tailored prevention strategies and support advocacy in low-income settings in SSA.

The specific objectives were to: i) identify CVD risk perceptions in different SSA settings (rural and urban) using citizen science mobile-phone EpiCollect application and photo-voices; ii) train citizen scientists in each project community on data collection, data assembling, data analysis and results presentation for advocacy purposes; iii) conduct citizen science-informed stakeholder advocacy workshops led by trained citizen scientists aimed at presenting results, identifying the key challenges, prioritizing tailored interventions, and co-design actionable steps to support CVD risk prevention.

## Methods

### Theoretical framework

**Fig. 2:**
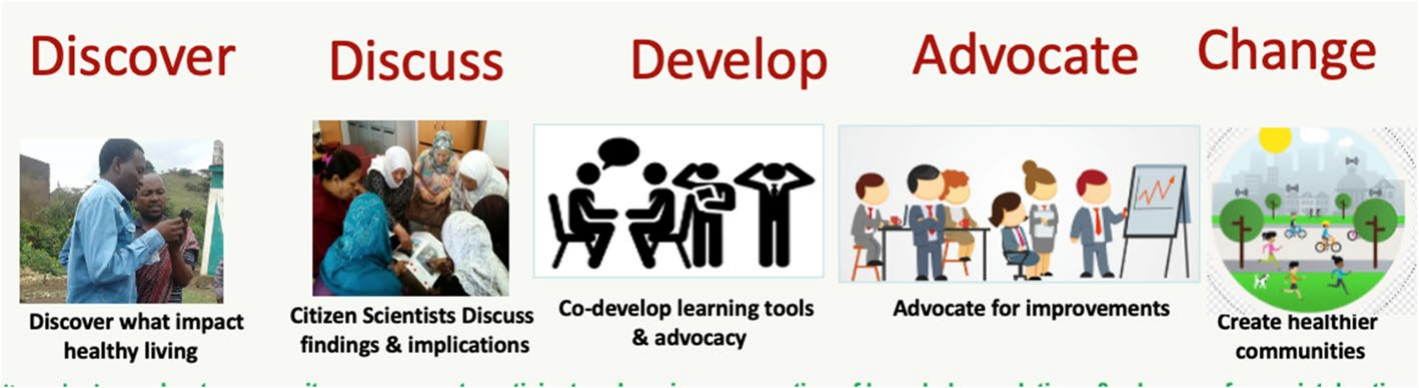
Adapted (‘Our Voice’) Citizen Science model

The concept of collaborative citizen science is based on the principles of both participatory action research and citizen science [25, 26], which has been widely used in public health, education, community development, agriculture and social work [26]. Collaborative citizen science is seen as a transformative process whereby researchers work with multi-level stakeholders (including community members, literate/non-literate citizens, decision makers, etc.) and the study participants to co-create knowledge and develop a sense of their community through engagement, and participatory learning. Collaborative citizen science emphasises mobile-based data collection, data analysis, interrogation of findings, prioritizing intervention(s) and advocating for a change at community-level. We adapted the “*Our Voice’ Citizen Science for Health Equity”* Stanford University citizen science model as a guide to explore (i.e. discover and discuss) CVD risk perceptions, and support participatory learning, co-development and advocacy for change [10]. The adapted ‘Our Voice’ model (*Discover, Discuss, Develop, Advocate, and Change*) is depicted on (Fig. 2.) In addition, integrated knowledge translation (IKT) and a robust community-specific stakeholders’ advocacy workshop activity with stakeholders [27, 28], were utilized to support participation, community engagement and involvement of multi-level stakeholders. The IKT approach which was intended to enable systematic, continuous engagement with relevant decision-makers throughout the research project, was a key feature of the CEBHA + project [29, 30].

### Study design and study population

**Fig. 3:**
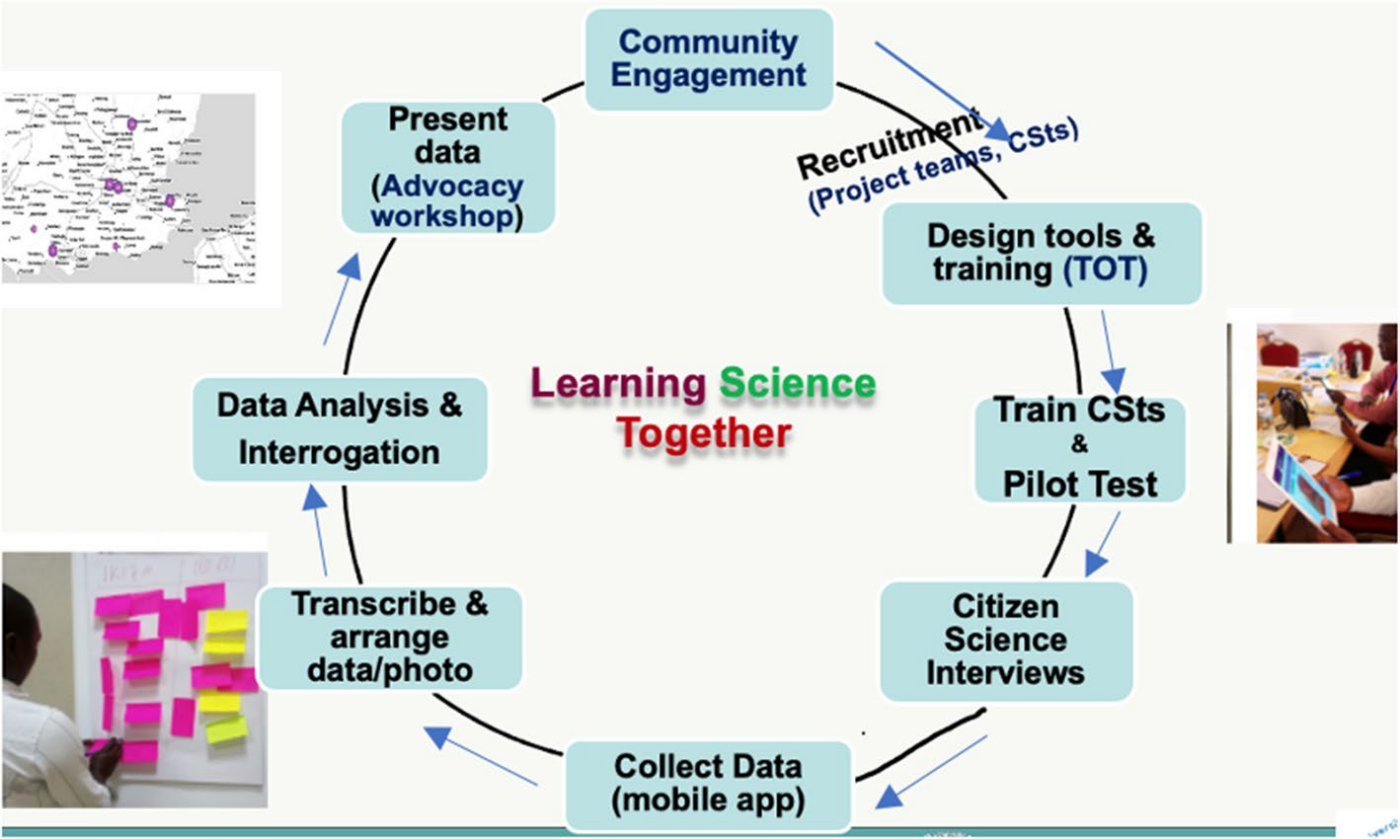
Citizen Science steps (schema)

**Fig. 4:**
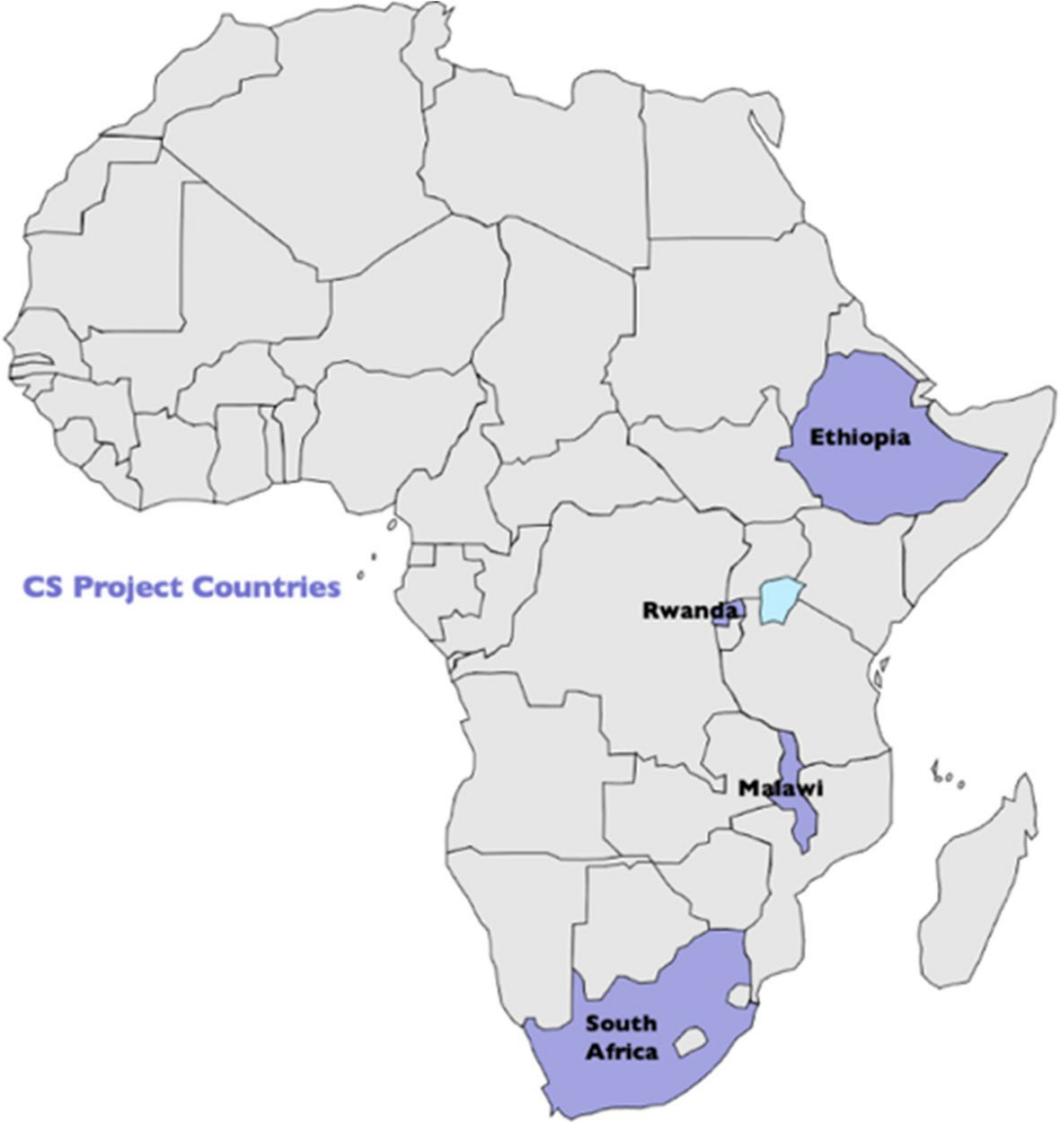
Map showing the Project countries

This study used a citizen science and participatory approach. The detailed methods are described in our protocol paper [24]. The schematic framework for which the study design was operationalised is presented in (Fig. 3.) (see also Table 1). This framework was further refined following our implementation in the Rwandan sites. The study was conducted in four of the five countries of the CEBHA + consortium, viz, Malawi, Ethiopia, Rwanda and South Africa (see map in Fig. 4).

**Table 1:** Citizen Science co-designing process with stakeholders: case of Rwanda adaptation [31]

Steps	Activity	Purpose	Number and categories of stakeholders	Total^a^
1	Consultation meeting	Sampling study sites	1 researcher and 1 staff at national level (MoH / RBC)	2
2	A three-day workshop	Discuss CEBHA + research Study Protocol with key stakeholders – for feedback	7 researchers, 2 project staff, 3 staff from different ministries, 1 staff from parliament, 2 staff from National Policy, 2 staff from civil society, 1 staff from city of Kigali, 1 academic from University of Rwanda, 1 staff from national referral hospital	20
3	Community engagement	Introduce study to local authorities, PHCs at district/sector levels; recruit citizen scientists	8–10 PHC stakeholders / local community leaders. Researcher had 8 meetings, each with different	10
4	A one-day meeting	Introduce the study to local community stakeholders	5 Researchers, 1 staff at MoH/RBC, 3 staff at district level, 15 PHC level, 53 local community authorities (4 executive secretaries at sector level, 14 executive secretaries at cell level and 35 chiefs of villages)	68
5	Recruitment of citizen scientists	Identify and select community member	1 Researcher and 2 health center staff	3
6	Training of the trainers on citizen science	Train researchers on citizens science /data collection	5 Researchers (including one researchers who trained his fellow researchers) and 2 project supporting staff	7
7	Citizen scientists training (session 1 & 2)	Train citizen scientists on data collection, analysis and advocacy	7 Researchers, 3 supporting staff, 12 community members (citizen scientists-to be), 4 staff from HCs	22
8	Feedback meeting I	Sharing findings from a pilot citizen science	6 Researchers, 2 project supporting staff, 18 local community members, PHC stakeholders (6 HC and 12 CHWs coordinators, 12 citizen scientists)	26
9	Citizen science data analysis meeting	Data analysis by citizen scientists	7 researchers, 2 project supporting staff, 12 citizens scientists	21
10	Feedback meeting II	Sharing and validating preliminary findings from citizen science data	5 researchers, 2 project supporting staff, 3 staff at ministry level (MoH/RBC), 16 KUs at local community level (4 health center staff and 12 CHWs coordinators) and 12 citizen scientists	26
11	1-day community advocacy workshop in each of urban & rural study site	Sharing findings and advocating for action to prevent CVDs	6 researchers, 2 project staff, 3 stakeholders at MoH level, 1 stakeholder at district level, 4 local community leaders at sector level, 12 local community leaders at cell level, 18 staff at PHC level (6 HC staff and 12 coordinators of CHWs at cell level), 12 citizen scientists, 16 representatives of national youth & women councils, village chiefs	72

^a^Total number of stakeholders, *PHC* Primary Health Care, *MoH* Ministry of Health, *RBC* Rwanda Biomedical Center, *PHC* Primary health care, *NGOs* Non-governmental Organizations, *HC* Health center, *CHWs*-Community Health Workers

### *Setting and sampling of study participants*.

Two communities (rural and urban) were purposively selected in each country, except for South Africa, where two townships near the Cape Town metropolis were purposively selected for the study. In each community, 6–8 community members were recruited during community engagement and consultation meetings, and later trained as citizen scientists– with a total of 12–16 per country (See Table 2). The trained citizen scientists facilitated the recruitment of participants in their neighbourhood for the citizen science survey, using purposive and snowballing sampling techniques. The number of participants recruited and interviewed in each project setting by country are presented on Table 2.

**Table 2:** Description of the citizen science study in the four countries: participants, scope and immediate outputs

Project Parameters	Ethiopia		Malawi		South Africa		Rwanda		
***Communities***	Rural	Urban	Rural	Urban	Township 1^**+**^	Township2^χ^	Rural	Urban	Total
***Consultation meetings***	3	4	2	3	5	4	3	4	28
***Citizen Science EpiCollect Survey***									
* Trained Citizen Scientists *^*α*^	6 (3 M/3 F)	6 (4 M/3 F)	6 (3 M/3 F)	6 (3 M/3F)	8 (4 M/4F)	7 (3 M/4 F)	6 (3 M/3 F)	6 (3 M/3F)	51
*Age range of Citizen scientists)*	27–65	25–65	19–65	18–64	20–65	18–69	28–45	23–63	
*Number of Participants Surveyed*	25(15 M/10F)	21 (7 M/14F)	21 (9 M/13F)	22(8 M/14F)	41(11 M/30F)	27(9 M/18F)	24(10 M/15F)	24(11 M/12F)	205
*Education Level: None*	12 (48.0%)	2 (9.5%)	10 (47.6%)	12 (54.5%)	0 (0%)	7 (25.9%)	0 (0%)	0 (0%)	
*At least a Primary level*	13 (52.0%)	8 (37.9%)	11 (52.3%)	10 (45.5%)	41 (100.0)	18 (74.1%)	24 (100.0%)	24 (100.0%)	
FGDs/participants number*	2 (13)	2 (12)	2 (12)	2 (12)	7 (76)	4 (38)	4 (34)	3 (31)	26 (228)
***CS Results***									
Number of photos taken	125	105	105	120	142	120	~ 130	148	995
Number of narratives recorded	150	126	126	132	193	180	135	130	1172
Reported a relative with CVD (Yes)^#^	10 (40.0%)	18 (85.7%)	15 (71.4%)	18 (81.8%)	21 (50.0%)	17 (56.7%)	280	294	129(63%)
Willing to visit clinic if screened and referred by CHW^	25 (100%)	21 (100%)	20 (95.2%)	22 (100%)	41 (100%)	27 (90.0%)	20 (83.3%)	16(66.7%)	83–100%
**Advocacy & Prevention Strategies**									
Integrated knowledge translation activity	Yes	Yes	Yes	Yes	Yes		Yes	Yes	
Number of advocacy workshops held	1	1***	1	1***	1**		1	1***	7
Stakeholders^$^ in advocacy workshop	45	48	38	43	45		56	45	320
Organizations stakeholders come from ^**!**^	MoH, E, T, cardiovascular disease, C		MOH, E, T, C		MOH, E, T, H, C		MoH, E, T, H, R		
Stakeholders discussed/prioritized CVD prevention strategies	Yes	Yes	Yes	Yes	Yes	Yes	Yes	Yes	

^*^ The study participants were men and women aged 18 and above who were residents of the study community; most had limited education (high school of primary education)

^*α*^ ^ Citizen scientists' levels of education in the countries: Ethiopia (Grade 3–12); Malawi (Grade 4-); South Africa (Grade 6–12; Rwanda (Primary 3-High school); **A combined advocacy workshop was held in Cape Town; *** One (1) advocacy workshop & one (1) IKT workshop with decision makers; ^CHW – Community Health worker or volunteer; ^$^Stakeholders here included the citizen scientists; ^#^ Heart-related diseases (or CVD) such as heart attack, heart failure, stroke, myocardial infarction, angina, and hypertension; ^**!**^Community, district and national-based policy-level stakeholders, including leaders in the Ministry of Health (MoH), Education/academia (E), traditional/Religious (T), Local Health Committees (H), Rwanda Biomedical Centre (R), Village Chief/head (C), etc.) who participated in the advocacy workshops; + Black South African-dominated township; ^χ^Coloured South Africa-dominated township

### Recruitment of Citizen Science survey participants

The inclusion criteria for participants were i) men and women aged 18 years and above who lived in a project community for more than 2 years; ii) those with one or more CVD risk factors (e.g. tobacco smokers, persons with hypertension, obesity, and diabetes); and iii) those with no known CVD risk factors but have possible exposures to risk factors. In addition, the participants recruited were mainly those that resides within 100–120 m radius from the citizen scientists home or location. Each trained citizen scientist recruited 3–4 participants from his/her neighbourhood after giving a description of the study and receiving verbal consent to participate. An average of 26 participants were recruited in each of the eight communities, thus meeting our targeted sample of 54 persons/country. The main focus was on those with at least one CVD risk factor. Participants in Rwanda, Malawi and Ethiopia were sampled using a convenient sampling method, while those in South Africa were sampled using snowball sampling technique (i.e. participant identify other potential participants for participation. To ensure that the recruited population was representative of the larger population, the project teams in each country ensured that citizen scientist were recruited from a wide-spread clusters or locations in each rural or urban setting. The citizen scientists were trained on the use of the EpiCollect mobile app to collect data from the participants as per the study protocol (23). A standard operating procedure (SOP) was provided also to each citizen scientist on what, how and when to take a picture and narratives (phot-voices).

*Citizen Science* (EpiCollect) *questionnaire development and data collection.* In each country, the project team and the citizen scientists supported the development, revision, and translation of the EpiCollect-based questionnaire used for the survey. The 6-item survey questionnaire was pilot-tested in the neighbouring communities and translated into local dialects before use. The questionnaire was adapted for each country based on its context; it had four sections viz: i) demography (viz. country, location, age, and gender); ii) perceived importance of the heart iii) CVD risk conception and perceptions, iv) exposure and perceived threats of CVD/risks, v) options of communication of CVD risk in the community; and vi) health seeking intentions.

The EpiCollect mobile app (https://five.epicollect.net/) was adapted as a multi-dimensional mobile platform and used to collect participants questionnaire survey data as well as photos and narratives (i.e. photo-voices); capturing local environmental features related to cardiovascular health [32]. There was no financial compensation given to participants recruited for the study, as citizen scientists were trained to interact with neighbours who were willing to participate voluntarily. However, the citizen scientists were provided a stipend (transport and meals) for 3–4 days when they engaged in the fieldwork.

### Project implementation and adaptations

This study used systematic and community-engaged processes to support participation of multi-level stakeholders. The key steps we followed in implementing the study are outlined in our framework (Fig. 3). The detailed specific activities we implemented (as informed by our framework and co-design process) included i) community engagement, ii) recruitment and training of community members as citizen scientists, iii) co-designing of tool/questionnaires, iv) citizen science interviews, data collection and analysis/interrogation, vi) qualitative inquiry (focus group discussions); and vi) stakeholder advocacy workshops. These steps have been previously described in our participatory citizen science project protocol paper [24]. The projects were implemented just before, partly during and after the COVID-19 pandemic (2019–2023), and had some modification based on country-specific COVID-19 regulations. The community engagement activity, citizen science survey, and advocacy workshops were undertaken in two of the countries (viz. Rwanda and Ethiopia) by March 2020. Following the onset of the pandemic, the project teams (i.e., the coordinating office and the country-level teams) met virtually to adapt project delivery processes in line with the pandemic regulations in the countries and to support implementation fidelity. The summary adaptions made and the implementation processes and outputs of the projects in the countries were as follows:

i. Community members/stakeholders’ engagement. A multi-sectoral stakeholder engagement process was implemented in each country to facilitate the learning and co-creation. The engagement process was undertaken more substantially in Ethiopia and Rwanda (as community engagements were done before the COVID-19 pandemic lockdown) compared to Malawi and South Africa where engagements and advocacy were implemented after the lockdown. The citizen scientists were recruited during community engagement phase (See Table 1).
ii. Qualitative enquiry. Prior to launching the citizen science survey, each country conducted 6–11 focus group discussions (FGDs) with community members. This was aimed as an initial qualitative inquiry to understand the community and individual’s perspectives on CVD and associated risk factors in poor SSA rural and urban settings. Although FGDs were conducted as part of the overall study, this paper focuses on the co-design activities implemented, and the data collected on Citizen Science using EpiCollect questionnaires. The FGDs are outside of the scope of this paper, and detailed steps and results of the FGD are reported in previous papers under our qualitative enquiry [32, 33]. The preliminary results from the FGDs informed the conducting of the citizen science survey in each country.
iii. Co-designing activities. Using consultation meetings, planned workshops, and community engagement, the citizen science and co-design process was implemented. This involved i) engaging with stakeholders to learn about cardiovascular disease, its epidemiology and implication, ii) recruiting community members with skills on community advocacy; iii) working with project teams to design questionnaire for Citizen Science interviews (photo-voices); iv) Data collection, analysis and interrogation of findings; v) listing key finding and the implications; vi) prioritizing tailored interventions strategies and discussing implementation.

Fifty one (51) trained citizen scientists facilitated the co-designing process with stakeholders and the research team. They also supported data collection and analysis, and supported the advocacy workshops involving stakeholders in each community. In Rwanda, for example, the citizen scientists and the stakeholders (considered as data users) were involved in planned co-designing activities to achieve set integrated knowledge translation (IKT) results as seen in Table 1 [31]. Findings from the Rwandan arm of the study had informed the implementation of the study in the other countries.

iv) Stakeholder Advocacy Workshops. A total of seven community-specific advocacy workshops were held in the participating countries near the end of the project. The purpose of the workshops was to present results of the study to community members, the decision-makers and other stakeholders in each country, to discuss the implications of the study findings and advocate for community-level strategies to prevent cardiovascular disease. Prior to the advocacy workshops, the country-level project teams consulted, and engaged with relevant stakeholders with the aim to introduce the project, recruit citizen scientists, and call for participation in advocacy meeting [34]. The guidance for organizing the workshops was developed and is described in our published protocol paper [24]. Findings from the citizen science survey and summary of preliminary FGD results were used to guide the advocacy workshops.
v) Follow up advocacy for prevention intervention action. Following the stakeholder advocacy workshops in the communities, the project teams (including the principal investigators [PIs], researchers and stakeholders) in each country undertook follow-up engagement and consultations with the stakeholders. The essence of the follow-up advocacy was to further meet with key decision makers and stakeholders, such as the focal persons of NCD Units in the Ministry of Health (in Ethiopia), the Health Advisory Facility Committee (in Malawi), and the Rwandan Biomedical Centre to follow up on the agreed action plans and promises made during the stakeholder advocacy workshops. In South Africa, follow-up advocacy and feedback meeting was held with key community leaders, citizen scientists and stakeholders in health sector and academia.

### Data preparation and analysis

**Fig. 5:**
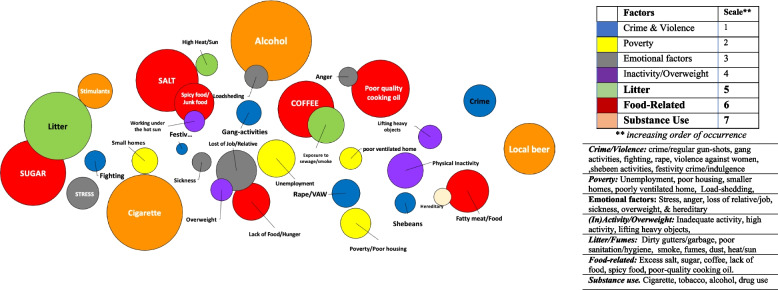
*A: Perceived causes (and risk factors) of cardiovascular diseases in South Africa, Malawi and Ethiopia (combined) *Bubble is calculated as weighted proportion of each factor over the overall total for identified factors

**Fig. 6:**
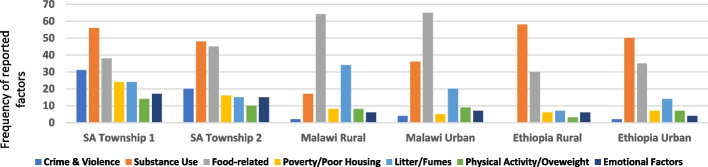
Perceived causes of *heart-related diseases* in six community settings* * Bar chats were plotted using frequency of occurrence of specific factors (as reported) per community in each country. The frequencies were not weighted by the overall total for all group

**Fig. 7:**
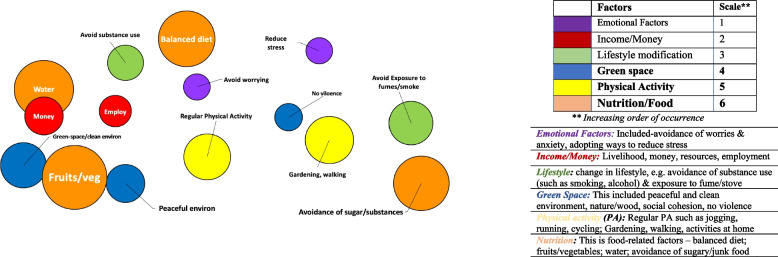
Perceived positive (mitigating) factors for *heart-related diseases in South Africa, Malawi and Ethiopia (Combined)* * Each bubble is a weighted proportion of each factor over the overall total for the identified factors

**Fig. 8:**
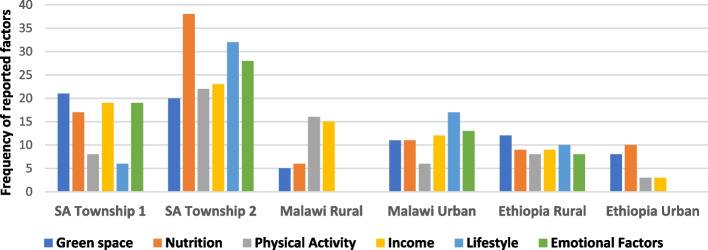
Perceived mitigating factors for *heart-related diseases* by community/settings (Bar graph) * Bar chats were plotted using frequency of occurrence of specific factors (as reported) per community in each country

Data from the four countries were analysed for the study. However, one aspect of the EpiCollect data (i.e. photo-voices on CVD risk perceptions for Rwanda), was not available at the time of data pooling and analysis. For this reason, our (Figs. 5, 6, 7 and 8.) (see results section) are presented for the combined data from three of the four countries (i.e. Ethiopia, Malawi and South Africa). The country-specific data collected by the citizen scientists were first extracted from the EpiCollect platform and organized by the research team and the citizen scientists from each community in the respective study countries. Simple thematic analysis methods were adopted and included discussing individual narratives and the photos in groups of 3–4 citizen scientists and researchers [35]. Then with support from the research teams, the citizen scientists summarized the priority issues and documented these using cardboard and flip-charts in each project community. Descriptive analysis, as well as thematic and comparative analysis were undertaken by the citizen scientists and the lead researcher (KO) to describe and compare CVD risk perceptions in and across the project sites and countries. The findings were summarized and made available for presentation by designated citizen scientists during the advocacy workshops. In addition, the data on perceived causes and mitigating factors for CVD were analysed with the help of citizen scientists, and presented by KO (first author) using bubble graphs and bar charts. Themes were first determined, and then streamlined to 6–7 key factors per setting/country. The frequencies of occurrence of specific factors (as reported) per community in each country were determined. These frequencies were then weighted by the overall total (count) for all groups in the combined data for the countries. The ‘bubbles’ in the bubble graphs (Figs. 5 and 6) were calculated as a weighted proportion of each factor (as reported by participants) over the overall total for the identified factors in the three countries. The bar chats were plotted using frequency of occurrence of specific factors (as reported) per community in each country.

### Reflexivity

The researchers in the project countries led the research, and worked with the citizen scientists and the principal investigators of the project and identified stakeholders. Each country project team members had learned to respectively engage with the citizen scientists and stakeholders right from the planning and implementation stages onto the end of project without issues and negative interferences. The participatory learning, community engagement/involvement activities seemingly supported cordial and friendly relationships between the citizen scientists, research teams and community members.

## Results

### Project outputs and outcomes

The key outputs and intermediate outcomes of the project are listed in Table 2. About 28 consultation meetings (3–5 in each community) were held with the community stakeholders as part of the initial community engagement activities. Fifty one citizen scientists (52% of these were women), were recruited and trained to lead the citizen science activities including community advocacy workshops. We collected 100–150 photographs and 150–240 voice recordings on CVD risk perceptions, communication and health-seeking intentions. A total of 205 participants were interviewed by the trained citizen scientists. Nearly three in every five (63%) of the participants interviewed reported having a relative with a CVD.

Over 90% of participants were willing to visit a nearby health clinic for re-assessment if screened and referred by a community health worker. In each country, multi-level stakeholders (ranging from 81–101 persons) attended the advocacy workshops conducted, and discussed and agreed on prevention strategies. The results of the citizen science study and advocacy workshop activities are presented below. In addition, 26 FGDs) were conducted during the qualitative enquiry in the rural and urban communities. The FGDs findings (for Rwanda) have been published elsewhere [33].

### Co-designing and integrated knowledge translation

In all the countries, the project teams worked hard to embed citizen science into their CEBHA + -supported integrated knowledge translation (IKT) strategy, resulting in robust stakeholders’ engagements, data demand and information use, and policy dialogue [31]. Specifically, in Ethiopia and Rwanda, our collaborative citizen science project proved to be an important strategy for research co-design and solution co-production. In these two countries compared to the others, the citizen scientists (data collectors and users) and the decision makers (considered as data users) were highly involved in planned co-designing activities towards achieving IKT results. Implementation of IKT strategies were implemented alongside the citizen science study but within a different research work package (Evidence-based Public Health) [23].

### Perceived risk factors for cardiovascular diseases (photo-voices)

Photo-voice data on perception of CVD risk were available for three of the four countries (i.e. Ethiopia, Malawi and South Africa). The participants in these three countries indicated that their main perceived causes of CVD (such as hypertension, stroke, heart failure, heart attack, and angina) were *substance use* (cigarette smoking, excess alcohol and drug us), *food-related factors* (excess salt, sugar, coffee, lack of food, and poor-quality cooking oil), and *litter*, in that order. These were followed by physical inactivity, emotional factors, poverty, and crime and violence (Fig. 5). A detailed community-specific analysis of these factors is presented in Fig. 6 (bar charts). Notably, in the two South African urban townships, substance abuse, crime, and litter were the most commonly mentioned risk factors relative to the other countries (Fig. 6).

- i) Substance abuse and crime:

High intake of alcohol was considered a risk factor across all countries – though the type differed by country. The types of local alcohol in the countries were *Areke* (local gin in Ethiopia), *Farso* (local beer in Malawi) and *Kachasu* (local spirit in Malawi). In addition, chewing *khat* (local stimulant) in Ethiopia and *nyaope (i.e. mixture of heroin, cannabis products, antiretroviral drugs, *etc.*)* in South Africa were considered risk factors for CVD in both the rural and urban areas. The concerns around smoking, drugs and violence were mentioned in most urban communities. In South Africa (Township 1) for example an older woman stated this:

“Those who are smoking nyaope in our community can grab (i.e., kidnap or rape) our kids and also break into our houses. These situations usually bring anxiety resulting in high blood pressure – and makes one to be at high risk of heart attack. [South Africa, Township– 1—65-year old woman].

- ii)Food-related factors***:***

The perceived effects of intake of excess sugar, salt, and poor quality vegetable oil on the heart was emphasized in the countries. Examples of these assertions are given below:

“Excess sugar, salt, coffee and alcohol (Kachasu) are very bad for the heart and body. Also, the lack of food (at home) can cause malnutrition, and stress – these affect my heart”. [Malawi, 49-year old woman -urban].

“Much oil (from poor quality vegetables oil) accumulates around the heart and blocks blood vessels thereby causing heart disease and paralysis”. [Ethiopia—42- year old woman -urban].

- iii) Poverty, crime and violence:

Participants interviewed in South African townships, and urban Malawi, Ethiopia and Rwanda emphasized the notion that poverty is one thing that aggravates substance abuse, crime and violence in their communities “.

"The community where we are living is a slum. Most people are unemployed, and poverty is high so people tend to drink a lot, and crime is very high. [Malawi—35-year old –an—urban].

Others commented also on violence and crime as follows:

“There are lots of crimes going on in our community, and this is very dangerous to our lives. The sound of gunshots can lead to heart attack” [South Africa—58- year old wo–an—Township 1].

- iii) Litter (poor sanitation and hygiene):

The problem of litter was highlighted in all the communities and countries surveyed.

“Unclean environment, stagnant water without proper toilet is bad for health especially to our children and the elderly in the community. [Ethiopia—25-year old wo–an—urban].

“Litter is a high risk. This is because we get to inhale all the bad things that come from the dirt, and that can make us get sick”. [20-year old female SA Township 1].

### Perceived positive factors for cardiovascular health

The positive factors for cardiovascular health as perceived by participants are represented in the bubble charts in Figs. 7 and 8. In a combined data available from three countries (Malawi, Ethiopia and South Africa), the main factors that participants in all communities indicated as those that will help mitigate their cardiovascular health risk were *nutrition or food-related* (i.e. balanced diet, fruits/vegetables, water), *physical activity*, and *green space* (i.e. clean environment, green space, and peaceful communities), in that order.

Other mitigating factors mentioned were *lifestyle modification* (e.g. stopping smoking, and tobacco and alcohol use, and avoidance of exposure to fume/stove); *having income* (money or resources), and dealing with *emotional factors* (such as stress, worry and anxiety). *“Exercise, and gardening are very good for your heart (mind) and spirit. It is engaging, and you feel happy afterwards …”* [South Africa – 64-year old man – urban township].

“Where there is gentle breeze blowing (i.e. green nature space), and no no–se—a person will have a peaceful life, and cannot suffer from heart disease because this is calming, and make one feel good. [Malawi—50-year female-Urban].

Figure 8. shows the distribution of the reported positive factors (and frequency of occurrence) by community, with *nutrition, green space*, and *income* being three major factors reported in South African townships, and the rural and urban communities of Malawi and Ethiopia. In all the countries, participants indicated that a peaceful and natural environment (green space), and physical activity as factors that affect the heart positively.

### Identified barriers, and prioritized CVD prevention strategies and actions

The seven most identified problems or barriers to CVD risk prevention and the prioritized prevention strategies agreed upon during the advocacy workshops are presented in Table 3. In all the countries, poor perception, knowledge and awareness gap with regard to CVD and their risk factors were recorded as key barriers to CVD prevention. The other barriers differed by country and by locations within countries. In all the rural and urban communities, poor perception and awareness of CVD despite high CVD risk in communities, litter/garbage (or poor sanitation/hygiene), limited access to CVD prevention services and high rates of substance use were the common and priority problems that needed to be addressed. There was a high degree of similarity in the prevention strategies agreed upon by the stakeholders in most of the countries.

**Table 3:** Advocacy workshops results: Identified problems/barriers and prioritized prevention strategies and actions who’s responsible

Problems/Barriers	Country	Prioritized Strategies/Solutions	Who?	How?	Was it done?^a^
***Poor perception and awareness on CVD in communities***	Ethiopia(Rural/Urban)	Awareness campaigns in communities	MoH, AHRI, schools, faith-based	Use IEC/BCC materials for campaigns	Done
	Malawi(Rural Urban)	Conduct NCDs awareness campaigns at community level by trained CHWs	MoH, PHC Managers, HACs	Trained and deploy CHWs to support for community sensitization	Not done
	Rwanda (Rural & Urban)	Designing community-led participatory strategies to address knowledge gap	MOH/RBC—District level; Health committees in Sector, cell & village levels	Sensitize local communities on CVD and risk factors	Done
	South Africa (Townships)	Support awareness creation on CVD risk at schools, and townships	Health committees, district-level DoH	Involve Western Cape on Wellness (WoW) hubs to support CVD prevention campaign	Not done yet
***Physical Inactivity***	Ethiopia (Urban)	Promote and encourage physical activity in schools, workplace and community	Civil Service, community, PHCs, and village to support	Arrange physical exercise (weekly) events at different workplaces and communities	Not done yet
	Rwanda (Rural & Urban)	Create awareness, and provide access and support for physical activity initiatives	MOH, Health committees, volunteers, schools and religious centres	Organize PA events at work place, communities/village levels	This has been done
	South Africa (Townships)	Promote and encourage physical activity in schools, workplace and community	NGOs, Western Cape on Wellness (WoW) hubs to support PA in communities	Arrange PA events at community-level. Support open streets townships	Partly done by NGOs
***Limited access to community-level CVD prevention information & services***	Ethiopia (Rural & Urban)	Implement NCD/CVD risk screening and care intervention	AHRI CEBHA + & CDIA teams, in conjunction with MoH/NCD Unit	AHRI to train CHWs/HEWs to deliver CVD risk screening and referral to care	Done. Over 3000 persons screened
		Organizing training for CHWs/volunteers on NCD/CVD	MoH, Regional Office, Zonal Office, and Town Health Offices, AHRI NCD Unit	1. MOH/NCD unit to re-train and deploy more HEWs to communities. 2) Use citizen science	Done in collaboration with NCD unit (MoH)
	Malawi (Rural & Urban)	Community health promotion at community-level events	NCD Focal person, MOH, HAC. MOH	Build capacity and provide resources for NCD-related community health programmes	Not done yet
	South Africa (Townships 1&2)	Implement community-engaged CVD prevention health promotion & screening	DoH, WoW, Local health committees	Undertake CHWs-led NCD risk screening and care intervention	Was pilot-tested in 2 townships previous
	Rwanda (Rural & Urban)	CHW-led CVD risk screening and referral for care	Rwanda Biomedical center, primary health care stakeholders, project research team	Train & deploy CHWs for CVD risk screening, referral and care intervention in communities	Done
***Poor diet /junk food (nutrition)***	Ethiopia (Rural & Urban)	Community-based intervention targeting preparation of healthy diet from locally available food items	AHRI project team to support NCD unit with IEC/BCC materials on healthy food	MOH, NCD unit and AHRI CEBHA + team to support health promotion on health eating	Awareness programme held
	Rwanda (Rural & Urban)		MOH, community leaders, volunteers, Citizen scientists, schools and religious centres	Train Citizen scientists /volunteers to support preparation of healthy diet from locally available food items	Has been done
	South Africa (Townships)	Community-based intervention targeting healthy diet from locally available food	MOH, community leaders, volunteer, schools and religious centres	Train CHWs, and WoW members to support local training on food preparation and budget	Not done yet
***Dumping of cheap poor quality vegetable oil***	Malawi (Rural & Urban)	Facilitate access to good quality cooking oil, and ban inferior quality vegetable oil	Malawi Bureau of Standards, MOH, research and advocacy groups	Present/discuss Policy Briefs developed to Malawi Bureau of Standards	Policy brief developed
***Litter: Poor sanitation/hygiene***	Ethiopia (Rural & Urban)	Community health education to provide information on sanitation an hygiene	MoH, MoE, AHRI CEBHA + team	Collaboration with MoE, and MOH to support community participation in waste management	Not done yet
	Malawi (Urban)	Community-level support for adequate waste management & clean environment	MoE, MOH, PHC, community-based organisations, HAC	Commission community sanitary committee to support clean environment and households	Advocacy done with key stakeholders
	Rwanda (Rural & Urban)	Emphasising adequate litter and waste disposal	MOH, community leaders, volunteers, citizen scientists, schools, religious places	Sensitization of community members support sewage system maintenance	Has been done
	South Africa (Townships)	Support effective litter and waste disposal	Municipality to communities participatory, waste management initiatives	Sensitization of community members and villages to support weekly waste management	Not done yet
***High rates of alcohol use, cigarette smoking & crime***	Ethiopia (Urban)	Conduct community health education	Ministry of health, Regional health office,	Health information in the local languages	Partly done in Adama
	South Africa (Townships)	Youth-driven community-based intervention targeting behaviour change	DoH- municipal and district; local health committees, local committee groups	Engage early with young persons in schools, communities, on violence/crime prevention	Not done yet

^a^Was the prioritized solution or intervention undertaken during the project duration? Done means, a 'Yes’. If not 'Yes', it is either 'Partly done' or 'not yet done' *HEWs* Health extension workers; *HAC* Health Action Committee; *Citizen scientists* citizen scientists; *MOH* Ministry of Health; PHC: Primary Health Centre; *CEBHA + * Collaboration for Evidence-based Health Care and Public Health in Africa; *CDIA* Chronic Disease Initiative for Africa; *RBC* Rwanda Biomedical Centre; *MoE* Ministry of Environment/Ministry of Works; *IKT* Integrated knowledge translation

In all countries, community-level campaigns and participatory strategies were indicated as solutions for addressing the poor perception and awareness barriers. In the South African townships and urban Ethiopia, strategies to reduce the high rates of alcohol use, cigarette smoking and crime were commonly mentioned; the prioritized interventions were youth-driven community-based behaviour change interventions. In Rwanda, the prioritized prevention strategies against substance abuse include sensitizing the youth and community members on the effects of illicit drugs sales and drug abuse. The prevention strategies listed for poor nutrition had included community-based programmes to train community members on how to prepare a balanced diet using locally available foods. In Ethiopia and Rwanda, to address the identified lack of CVD risk screening and prevention services, community health workers as well as Health Extension Workers (CHWs/HEWs) were trained to conduct CVD risk screening and blood pressure measuring device and weight scales were provided. In general, over 4795 adults were screened and about a third referred for care for CVD risk using CVD risk score assessment tool in Ethiopia [*n* = 1300] [36], and Rwanda [*n* = 995] [37], and in Malawi [about 2500].

## Discussion

This study is among the first examples of a large scale citizen science project systematically implemented across multiple communities in several SSA countries. It leveraged co-design and community engagement approaches to generate rich, country-specific data to inform decision making targeting the prevention of CVD [24, 31]. The main findings were: i) the perceived causes of CVD in all the countries were similar, but participants unexpectedly emphasised other indirect factors (such as litter, poverty, substance abuse, crime, violence, stress, loss of job/relative) which were not consistent with most conventional causes of CVD (i.e., physical activity, diet, cholesterol, lifestyle, and hereditary factors). Notably, crime and substance abuse were major issues in South Africa relative to elsewhere; food-related factors was dominant in Malawi, food-related and emotional factors were dominant in Ethiopian communities.

ii. trained citizen scientists successfully facilitated co-learning and co-production activities besides data collection, analyses, presentation and facilitation of advocacy workshops;
iii. the positive factors perceived to mitigate effect of CVD were mainly nutrition or food-related, physical activity, and green space (i.e., clean and peaceful communities). These positive factors were directly connected with the reported perceived causes in each community; and; iv) through advocacy workshops and IKT activities, stakeholders in each country (especially those in health sector) had supported prioritization and begin to implement some locally-relevant CVD risk prevention solutions, including CVD risk screening and referral to care.

In our study, we had observed that learning together and engaging community members in different settings as citizen scientists enabled the researchers and communities in identifying the most needed and meaningful science-enabled activities of relevance to these sub-Saharan countries. The results of this study indicate that multi-country community-driven citizen science projects can facilitate effective multi-level engagement and participation of community stakeholders (including both community members and policymakers) in exploring CVD perceptions and supporting the co-creation, co-development, advocacy and implementation of contextually relevant health interventions in SSA [15, 38]. The summaries of the implications of the main findings are discussed below:

### Perceived cardiovascular disease causes and mitigating factors by setting

The concerns around CVD and the perceived risk observed extended beyond the conventionally known risk factors (diet, physical activity, drug and substance use, etc.) in all the countries. These (additional) perceived factors included poor sanitation/hygiene, litter, crime, emotional stress, poverty, poor quality cooking stoves, and unrest/fighting. Consequently, using the findings, the citizen scientists and stakeholders (during advocacy workshops) had proffered solutions that were considered community-relevant to address these perceived extra-causes, particularly in the urban sites in South Africa, Malawi, and Ethiopia. Based on these findings, community-specific health promotion and education intervention were commonly indicated as preferred prevention strategies (see Table 3). A more detail findings on the CVD causes, mitigating factors, and their implications in each country and settings is documented in another paper under review for publication (Okop et. al.- forthcoming).

### Citizen Scientists facilitated collaboration, co-learning and co-creation

Through this study, communities (and citizen scientists) were empowered to collaboratively engage in science to: i) explore the perceptions and communication of CVD risk in their setting; and ii) support advocacy for CVD risk prevention using the evidence generated from the collaborative approach. It is believed that the collaborative approach facilitated co-leading of this multi-setting research project that helped foster an authentic partnership between the formal scientific community and groups of community members and stakeholders, as opposed to having the researchers try to "go it alone" without the indigenous context learning, planned researcher support, training, and co-production [14]. Participatory methodologies have been very useful in the co-creation, co-production and evaluation of public health interventions [39, 40]. The citizen-engagement, co-learning, co-design processes helped in eliciting knowledge and learning that were acceptable and trusted by the community to assist in the development of relevant and more meaningful CVD risk communication and prevention strategies. For instance, in all four project countries, an important barrier to prevention of heart-related diseases was found to be the poor perception, knowledge and awareness gap with regard to CVD and their risk factors. Notably, community members and stakeholders recommended interventions in each community to address the poor perception and awareness gaps in both the rural and urban communities.

Importantly, the trained citizen scientists gained transferable scientific skills, including hands-on training in conducting community-based surveys, use of mobile devices for systematic interviewing and data gathering, data extraction methods, simple analysis (compiling the findings in a usable, meaningful format), and presentation of findings as part of the stakeholder advocacy workshops. A majority of the citizen scientists shared their utmost excitement at being given an opportunity to learn to lead research and prevention advocacy activities as “community scientists”. They described their feelings of personal satisfaction and fulfilment as “local scientists” capable of engaging in community-driven indigenous science through anecdotal reporting from their project teams and during stakeholders’ workshops.

### Collaborative prioritization and implementation of locally-relevant solutions

Through advocacy workshops and IKT activities, stakeholders in each country were able to support the prioritization and implementation of locally-relevant solutions for CVD risk prevention. This approach helped empower communities to take action to improve their health and wellbeing by first taking a lead in exploring CVD risk, collecting and analysing data, identifying, and prioritising community-based strategies, and, finally, mobilizing support and advocacy for sustainable solutions [15]. For example, in Ethiopia (rural and urban), following community advocacy workshops, the stakeholders in collaboration with the Ethiopian AHRI (Armeur Hansen Research Institute) project team supported the re-training of 12 health extension workers (HEWs) on blood pressure screening and CVD risk screening. The trained HEWs conducted a 4-week CVD risk screening intervention programme in 10 communities using a mobile app. The persons who were identified as high risk for CVD (124 out of 3000) were referred to designated clinics for care. Besides Ethiopia, CVD risk screening and referral to care interventions were successfully implemented in Rwanda and Malawi. In South Africa, community-engaged CVD risk prevention health promotion and screening programme were identified as an essential strategy for addressing CVD prevention in the townships.

It was a great achievement to see some of the suggested community-level prevention intervention strategies (i.e. nutrition, physical activity, and sanitation and hygiene) being planned and implemented in Ethiopia and Rwanda following the stakeholders’ advocacy workshops in these countries.

### Previous studies and the methodological gaps

The use of citizen science and co-design approaches in developing interventions in different fields is growing, particularly in develop countries. In the context of adopting these approaches to CVD prevention interventions in multiple SSA, literature from SSA is, however, scarce. A recent study conducted in Birmingham, UK had showed that citizen scientists participated in over 12 technology-enabled assessments and supported the identification of urban features impacting age friendliness [39]. They also utilized that findings and engagement to co-produce recommendations to improved local urban areas towards active aging in urban settings. The above study is akin to our study. However, our study focused on multiple urban and rural settings in four countries, and not just on one particular city.

Although the community-based interventions are being implemented, there is a gap of non-collaborative and or non-participatory intervention approaches. For instance, a recent systematic review had reported that community-based interventions had successfully improved knowledge and create awareness on CVD and risk factors and influence physical activity and dietary practices in the developed country communities [4]. However, although most of these interventions were delivered by healthcare workers, CHWs, and volunteers, the studies did not specifically incorporate the citizen-engaged (citizen science) participatory approach in their delivery. Thus, resulting in the lack of co-designed evidence-based interventions that are tailored to the individual settings/communities. Also, these interventions were mainly conducted outside of Africa. Another recent review reported on patient active involvement and the use of m-health in supporting physical activity and co-production of health policies aiming at CVD prevention. Findings from that review indicated that patients (and beneficiaries) participations in interventions co-design process, though recognized as fundamental for CVD prevention, were lacking [20]. Our study had employed citizen science and co-design approaches to facilitate multi-level stakeholders’ engagement, participatory learning, adaptation of tools, and support co-creation of knowledge, and advocacy for social action. This is akin to the study conducted by Wallersteine et. al, and those indicated by Boeder and colleagues [21, 22], except that their studies were not undertaken in multiple settings. Importantly, our study had also involved multi-level stakeholders including decision-makers and community members from the onset of the research processes, and in prioritizing evidence-based interventions and implementation actions. Our study, therefore, closes the methodological gaps in designing and implementing community-based participatory citizen science projects in multiple low-income settings.

### Challenges and lessons learnt

This research, the first of its kind in exploring CVD risk perception and co-developing prevention strategies and advocacy in SSA settings, adds value to citizen science research and methodology globally. There were, however, challenges encountered during the study. While the initial meetings of the research teams and consultations with stakeholders in each country took place before the pandemic, the COVID-19 restrictions heightened the challenge of meetings timely in all the countries during the co-designing and implementation of projects. It was expected that much of the collaborative citizen science research activities needed to be facilitated by the beneficiaries (citizen scientists and stakeholders) but, due to COVID-19, we experienced delays in scheduling timely consultation meetings with the stakeholders. As a result, the project team had to exercise a great deal of patience in re-scheduling meetings and being able to work with the citizen scientists and stakeholders at the times they were available. In addition, there were delays in some settings (particularly in the rural communities) in getting the citizen scientists (after data collection) to support organizing the narratives and photos, data analysis and results interpretation. We also observed that 2–3 days of training of the citizen scientists was not sufficient to build their capacity to support the data collection, organization and analysis, and presentation as expected. In some countries, we had to organise additional training sessions for the citizen scientists to further enhance their skills. Future studies of this type will need to pay particular attention to such constraints and continue to work on creative ways for mitigating them. The key lessons learnt include: i) ensuring active participation of citizens in exploring CVD perceptions, co-designing and implementing research can support participatory engagement for inclusive learning, co-designing, and co-creation intervention actions to address CVD prevention. ii) the process of producing reliable knowledge can be developed and enacted by citizens themselves with support from researchers; iii) importantly, working with teams across multiple countries and settings to support efficient research implementation and intervention sustainability requires dedicated resources, and adequate time allocation for team building and co-learning from the planning to evaluation stage.

#### Study strengths and limitations

This study used robust systematic and community-engaged processes to support participation of multi-level stakeholders in participatory research towards addressing local health problem with community-specific solutions. The collaborative methods supported active and productive citizen-led participatory research engagements. The study has some limitations. It was conducted only in selected communities in four countries in SSA, and therefore, the findings may apply in each individual project country, but may, however, not be generalizable in the SSA region. While the citizen science project was conducted in Rwanda the photo-voice data on CVD risk perception were not available for analysis. However, findings of this study can be utilised to support CVD prevention programmes in similar settings in SSA. It is important to note that the citizen scientists undertook the data collection, analysis, and outlined key findings that they prioritized for presentation to stakeholders during advocacy workshops. We, therefore, believe that the citizen scientists’ views on key issues, prioritized health problems and prevention strategies might be partly affected by their personal exposure, cultural perceptions, experience and learning.

### *Implication* for policy and practice

This citizen science study included citizen engagement, participation and involvement that conventional science often lacks. It emphasizes working with communities, volunteers, and multi-level stakeholders to support participatory learning and engagement to gain knowledge and context-based research perspectives that can impact society. Findings from this study have indicated the possibility of supporting the co-designing of CVD prevention strategies and actions in the context of multiple low-income settings in Africa. The collaborative identification of the community-level perceptions, barriers and facilitators of CVD prevention, and the subsequent prioritization and implementation of locally-relevant actionable solutions in the different settings are important lessons. Notably, the engagement with stakeholders resulted in the implementation of a pilot citizen science-informed CVD risk screening and referral to care project in three countries. This collaborative citizen science approach can be extended to other areas of public health to support the co-development of evidence-based solutions tailored to community needs.

## Conclusion

The collaborative engagement, participatory learning and co-designing approaches supported active engagement among citizen scientists, researchers, and stakeholders in exploring CVD and implementing context-specific insights to CVD prevention strategies in different SSA settings. We, therefore, advocate for the use of collaborative citizen science to foster learning and co-designing of community-based prevention and actionable advocacy strategies to address important public health problems (such as CVD and NCDs) in SSA settings.

## Data Availability

The datasets used and analysed during the current study are available from the corresponding author on reasonable request.

## Abbreviations

CVD: Cardiovascular diseases
SSA: Sub-Saharan Africa
LMICs: Low- and Middle-income countries
CHWs: Community health workers
PAR: Participatory action research
CEBHA +: Collaboration for Evidence-based Health Care and Public Health in Africa
NCDs: Non-communicable diseases
SES: Socio-economic status
HEWs: Health Extension workers
FGD: Focus group discussion
CDIA: Chronic Disease Initiative for Africa
